# Nutrition and metabolism in poultry: role of lipids in early diet

**DOI:** 10.1186/s40104-015-0029-9

**Published:** 2015-06-24

**Authors:** Gita Cherian

**Affiliations:** Department of Animal and Rangeland Sciences, 112 Withycombe Hall, Oregon State University, Corvallis, OR 97331 USA

**Keywords:** Chick, Docosahexaenoic acid, Early diet, Egg, Eicosanoid, Embryo, Essential fatty acids

## Abstract

Modern strains of broiler chickens are selected for fast growth and are marketed anywhere from 36 to 49 days after a 21-day incubational period. For a viable healthy chick, all the necessary nutrients required for growth and development must be provided by the hen through the fertilized egg. The current feeding strategies for improved growth, health and productivity are targeted towards chicks after hatching. Considering the fact that developing chick embryo spends over 30 % of its total life span inside the hatching egg relying on nutrients deposited by the breeder hen, investigations on nutritional needs during pre-hatch period will improve embryonic health, hatchability and chick viability. In this context, investigations on hatching egg lipid quality is of utmost importance because, during incubation, egg fat is the major source of energy and sole source of essential omega-6 (*n-6*) and omega-3 (*n-3*) fatty acids to the chick embryo. Due to the unique roles of *n-3* and *n-6* fatty acids in growth, immune health, and development of central nervous system, this review will focus on the role of early exposure to essential fatty acids through maternal diet and hatching egg and its impact on progeny in meat-type broiler chickens.

## Introduction

### Hatching egg: chick’s “early diet”

**Fig. 1:**
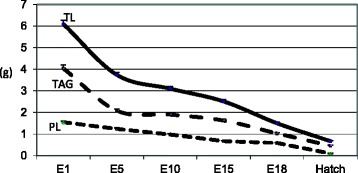
Pattern of total lipid, triacylglycerol and phospholipid transfer from hatching egg to the chick embryo during incubation*. TL = Total lipid, TAG = Triacylglycerol, PL = Phospholipid, E = embryonic age. *The weight of different lipid components in yolk or in remnant yolk sac (g) from day one of incubation through hatching period. n = 8

The hatching egg is a complex structure which provides physical and nutritional milieu to the embryo to sustain its growth into a healthy hatchling. The egg yolk or the “oocyte” is a single massive cell weighing about 17 ~ 20 g in an average egg and is comprised of 51–52 % water, 16–17 % protein, and 31–33 % lipids [1]. An average egg has over 5.5–6 g total lipids and is present as lipoproteins in the yolk. Among the total lipids, triacylglycerol constitute ~65 % of total lipids while phospholipids constitute ~28 % of total lipids in eggs. During the 21-day incubational period, over 88 % of triacylglycerol and 95 % of phospholipids are taken up by the growing chick embryo (Fig. 1). The rapid uptake of different lipid components by the embryo starts from the 2^nd^ week of incubation and continues till residual yolk is completely absorbed [2, 3]. Among the different lipids taken by the chick embryo, triacylglycerol serves as a source of energy while phospholipids serve as the essential structural precursors for membrane lipid bilayers [3]. Egg phospholipids are reservoirs of long chain (>20-carbon) polyunsaturated fatty acids (PUFA) such as arachidonic acid (20:4 *n-6*) and docosahexaenoic acid (DHA, 22:6 *n-3*). Through the provision of energy, essential fatty acids, PUFA and other vital nutrients (e.g., amino acids, antioxidants), nutrients in hatching egg serves as the first “meal” or the “early diet” of the developing chick embryo. Defects in nutrient supply during early life may have long term impact affecting growth, health, tissue maturation, as well as immune health of the progeny chicks.

### Breeder hen (maternal) diet and hatching egg lipid components

The physiology of the hen enables egg lipid and fatty acid manipulation within a short duration of time. Upon sexual maturity, hepatic lipogenesis is dramatically enhanced by estrogen in order to meet the demand for vitellogenesis. Yolk fats are synthesized in hen’s liver and are deposited to the yolk through serum via triacylglycerol-rich very low density lipoprotein (VLDL) and phospholipid-rich very high density lipoprotein vitellogenin [3, 4]. VLDL targeted towards yolk is about half the size of normal VLDL and is a specialized form of VLDL that is specific to laying hens called VLDLy [4]. VLDLy forms a complex with the ApoB_100_ and apovitellenin-1 (apoVLDL-II). ApoVLDL-II bound VLDLy molecules will not be acted upon by lipoprotein lipase (LPL), allowing triglycerides to be deposited to the oocyte intact [4]. No exogenous lipids are transported from the liver to the yolk, only *de novo* triglycerides are packaged into VLDL for transport. This allows for control over the fatty acid composition of the yolk.

### Enriching hatching eggs with essential *n-3* and ***n-6*** fatty acids

In chickens, α-linolenic acid (ALA 18:3 *n-3*) and linoleic acid (18:2 *n-6*) have to be supplied in the diet and are therefore called essential fatty acids. This essentiality is due to the inability of the hen to insert double bonds (due to the lack of desaturases) beyond δ-9 carbon and can occur only in plants. However, once a double bond is inserted at the 3^rd^ and 6^th^ carbon (from CH_3_ end locations), the hen can add more double bonds and form longer chain 20 and 22 carbon PUFAs. The process of long chain PUFA synthesis occurs mainly in the liver and includes Δ-6 desaturation, chain elongation and Δ-5 desaturation. Thus the parent ALA is converted to eicosapentaenoic acid (EPA, 20:5 *n-*3), which is subsequently converted to docosapentaenoic acid (DPA, 22:5 *n-*3) by chain elongation [5]. The final metabolite, DHA, is synthesized by chain elongation, Δ-6 desaturation, and peroxisomal β*-*oxidation of DPA [5]. Linoleic acid goes through the same pathway and conversion steps, with arachidonic acid being the major metabolite produced. The efficacy of long chain *n-3* PUFA from ALA depends on factors such as concentration of *n-6* fatty acids, because same desaturase and elongase enzymes are involved in the synthesis of *n-6* and *n-3* long chain PUFA. While both *n-3* and *n-6* PUFA share the same metabolic pathway, each family of fatty acids has been found to exert distinctly different and sometimes opposing biological effects.

In a typical breeder hen ration, linoleic constitute over 50 % of total fatty acids compared to ~3–3.5 % of ALA. This is due to the predominance of corn and the other sources of dietary fat that are high in *n-6* fatty acids. This imbalance in dietary *n-6* and *n-3* fatty acid is reflected in the absence of long chain *n-3* PUFA in commercial hatching eggs [6]. Oils from corn, sunflower, and safflower are rich sources of linoleic acids. In nature, there are limited sources of *n-3* fatty acid-rich oils that are economical and feasible for poultry feeding. Oils or oil seeds of flax (*Linum usitatissimum*), canola (*Brassica napus*), and chia (*Salvia hispanica)* are commonly used as *n-3* fatty acid sources in poultry diets*.* Among the different plant-based sources, flaxseed, owing to its high fat (>38 %) and ALA content (>50 %) along with other nutritional properties (e.g., metabolizable energy, protein) is the most common dietary ingredient explored to test the impact of breeder hen diet on egg *n-3* fatty acid content [7]. Hens fed flax incorporated predominantly ALA in eggs. Other marine sources (e.g., fish oil) also have been reported to enhance long chain *n-3* fatty acids (EPA, DPA and DHA) into eggs. Similarly feeding oil seeds rich in *n-6* fatty acids will lead to the incorporation of linoleic and other long chain *n-6* fatty acids such as arachidonic acid [7]. A list of some of the most common *n-6* and *n-3* fatty acids in hatching eggs and their concentration as affected by hens’ dietary lipid source is shown in Table 1. Due to the large turnover of lipids and metabolic pathways in egg laying hens, dietary fat composition is the major modifiable factor that affects the *n-3* and *n-6* PUFA composition of eggs and ultimately the chick embryo’s “early” fatty acid supply. In this context, it is noteworthy to say that the content and metabolism of *n-3* and *n-6* fatty acids in the hen diet and hatching egg is of particular interest and importance because of the actions of PUFA-derived metabolites (e.g., eicosanoids) in many biological processes in the hen and in the developing chick embryo and is explained elsewhere in this review.

**Table 1:** Polyunsaturated fatty acid composition of hatching eggs from breeder hens fed diet containing different lipid sources

Fatty acids, %	Fish	Flax	Palm	Sunflower	Corn	Safflower	Yellow grease
*n-6* fatty acid							
Linoleic (18:2 *n-6*)	7.8	10.4	8.7	19.7	9.3	21.5	17.4
Arachidonic (20:4 *n-6*)	0.6	0.8	1.6	2.1	1.5	2.2	3.1
Docosatetraenoic (22:4 *n-6*)	0.0	0.0	0.1	0.2	0.1	0.0	0.4
Docosapentaenoic (22:5 *n-6*)	0.0	0.0	0.1	0.1	0.1	1.0	0.0
Total *n-6*	8.4	11.2	10.5	22.1	11.0	24.7	20.9
*n-3* fatty acid							
Linolenic (18:3 *n-3*)	0.3	7.3	0.2	0.3	0.2	0.0	0.7
Eicosapentaenoic (20:5 *n-3*)	0.8	0.3	0.0	0.0	0.0	0.0	0.0
Docosapentaenoic (22:5 *n-3*)	0.5	0.1	0.0	0.0	0.0	0.0	0.2
Docosahexaenoic (22:6 *n-3*)	4.5	1.7	0.7	0.5	0.2	0.0	1.6
Total *n-6*	6.1	10.4	0.9	0.8	0.4	0.0	2.5

These oils or fat sources were added at 3–3.5 % in the breeder hen diets

The hens were fed an isocaloric (2,800 kcal/kg) and isonitrogenous (16.5 % CP) corn-soy-based diet and vitamins and minerals were supplied to exceed NRC requirements for breeder hens. The average weight of egg yolk in these trials were 16.5 - 18.0 g and the total fat of the yolk was 5.4- 5.6 g/egg

### *In ovo* nutrition through hatching eggs

*In ovo* supply of the embryo with vaccines is commonly done in poultry. Recently other substances (e.g., amino acids) injected into the hatching egg to boost metabolism and growth during early post-hatch period has been reported [8, 9]. However, such technology needs special facilities, time and capital to be adopted. Supplying the embryo with nutrients through “maternal” sources (e.g., breeder hen diet and hatching egg) is a natural and sustainable way to approach *in ovo* feeding. Using this concept, several studies were conducted in our laboratory to assess the impact of early exposure to lipids (e.g., essential fatty acids, conjugated linoleic acid, cholesterol) through hatching egg and its impact on tissue incorporation and fatty acid metabolism during pre and post- hatch period in meat-type broiler chickens [10–12].

### *In ovo* lipid nutrition and fatty acid changes in progeny chicks

**Fig. 2:**
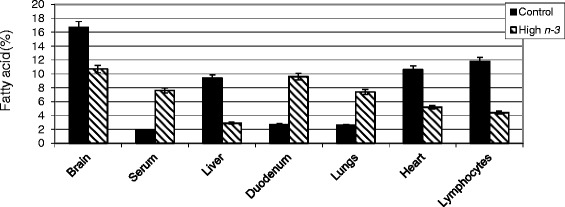
Total omega-3 fatty acids in the tissues or cells of chicks hatched from hens fed omega-6 (Control) or omega-3 enriched (High *n-3*) diet.**Control and High *n-3* represent maternal diet supplemented with 3.5 % sunflower oil or 3.5 % fish oil. Both diets were isonitrogenous (16 % crude protein) and isocaloric (2,866 kcal metabolizable energy). Total omeg-3 fatty acids include 18:3 *n-3*, 20:5 *n-3*, 22:5 *n-3* and 22:6 *n-3*

Alteration in tissue and cell membrane fatty acid composition of the embryo and the hatchling is the most significant impact of early exposure to lipids and essential fatty acids. This dramatic change in embryo and hatched fatty acid composition is brought about through yolk sac membrane (YSM), an extra-embryonic structure which grows out from the embryo surrounding the yolk during the early stage of incubation [13]. The avian YSM is a metabolically active organ. Recent studies from our laboratory on fatty acid changes during embryogenesis reveal that YSM serves as a reservoir of long chain 20 and 22-carbn *n-6* and *n-3* PUFA to the embryo [14]. To assess the impact of early exposure to lipids through hatching eggs on progeny chicks, feeds containing different oil or oil seeds (as sources of essential *n-3* or *n-6* fatty acids) were fed to broiler breeder hens. These fat sources include corn, sunflower, safflower oil (*n-6*, linoleic acid), flax, canola (ALA) or fish oil (EPA, DPA, DHA). Fertile eggs collected after 4 weeks of feeding were incubated. Tissues or cells collected from newly hatched chicks were subjected to fatty acid analysis. Significant changes in fatty acid composition of tissues and cells (e.g., brain, small intestine, cardiac, hepatic, spleen, bursa, lymphocytes) reflecting hen diet and yolk lipid profile was observed (Fig. 2). These results provide direct evidence to substantiate the role of “early” dietary exposure to *n-3* and *n-6* fatty acids through hatching eggs in modulating tissue or cell membrane PUFA composition in progeny chicks.

### Lipids in early diet and its impact on chick tissue fatty acid status during growth

For growth to occur, metabolic precursors must be available to the hatchling. Chicks are precocial and will forage immediately after hatching. However, management practices (e.g., transportation to farms, time gap in hatching window) limit early supply of nutrients to the hatchling through diet. For example, under practical conditions, the newly hatched chick usually does not have access to feed for over 48–72 h post-hatch [15]. Early feed deprivation post-hatch along with the absence of *n-3* PUFA in the current commercial hatching eggs may aggravate an *n-3* PUFA deficient situation in immune cells and vital organs. Moreover, long-chain *n-3* fatty acid in early diet plays crucial roles in immunity in the hatchling [10]. Long chain PUFA such as arachidonic acid and EPA serves as precursors for eicosanoids such as prostaglandins (PG), thromboxanes (TX) and leukotrienes (LT). Eicosanoids are lipid mediators of inflammation. Eicosanoids derived from *n-6* fatty acids are more pro-inflammatory than those derived from *n-3* fatty acids [16]. Therefore, establishment of a stable and sufficient cell membrane PUFA status during early life is critical for maintaining general metabolism and immune health of progeny chicks.

The impact of early exposure of lipids through hatching eggs on tissue PUFA composition in chicks during post-hatch was assessed. Eggs were produced by feeding breeder hens fish oil as source of *n-3* PUFA (*n-3* enriched) or sunflower oil as source of *n-6* fatty acids (*n-3* depleted). The total *n-3* fatty acids in *n-3* depleted or enriched eggs were 0.9 and 4.1 %, respectively [17, 18]. The chicks hatched from *n-3* PUFA enriched or depleted eggs were fed diets lacking in long chain (>20-C) fatty acids (simulating a commercial diet). The fatty acid composition of chick tissues were determined during the grow-out period. The chicks hatched from *n-3* fatty acid enriched eggs retained higher levels of EPA, DHA and total *n-3* fatty acid in the tissues and cells when faced with an *n-3* fatty acid deficient diet during growth. Similarly, the retention of arachidonic acid was higher in liver, heart, brain, spleen, duodenum and cells (thrombocytes, peripheral blood mononuclear [PBMN]) of chicks hatched *n-6* PUFA-enriched eggs [17, 18]. The efficacy of the tissues in retaining *n-3* or *n-6* PUFA varied among tissues and the type of cell membrane. For example, DHA content was higher up to day 14–28 of post-hatch growth in the liver, spleen, bursa and cardiac ventricle of *n-3* PUFA-enriched eggs when compared with those of *n-3* PUFA depleted eggs [19]. It is clear that early supply of high *n-3* fatty acids through egg offer certain advantage for offspring of *n-3*-enriched eggs, because they had more DHA available at post-hatch, which they obviously used during their first 14–28 days post-hatch. In the duodenum, we noticed that DHA content was highest up to d 14 of growth in chicks hatched from *n-3* fatty acid-enriched eggs [20]. A similar impact of egg lipid composition persisting up to 14 days post hatch has been reported in the bone cells in quails [21].

As acyl moieties of phospholipids in cell membranes, PUFA modulate membrane biogenesis, eicosanoid metabolism, and are essential for the optimal functioning of vital organs. In this context, effectiveness of pre-hatch vs. post-hatch supplementation of *n-3* fatty acids in enhancing tissue *n-3* fatty acid status in chicks were investigated. Hens were fed a high *n-3* (H) or low *n-3* (L) diet. Fish oil or sunflower oil was used as source of lipids in H or L diets. Chicks hatched from hens fed the H or L diets were raised on a high (H-H) or no (L-L) *n-3* diet. Thus there were 4 treatments (H-H, H-L, L-H, and L-L). In treatments where chicks received H-H diet, brain and hepatic DHA was higher than L-H up to d 20 and d 40 of growth [19]. Similarly, arachidonic acid concentration in brain and liver remained significantly lower in H-H chicks up to day 40 of growth. In conclusion, early supplementation of *n-3* PUFA through hen diet and hatching egg has a marked influence of progeny, regardless of post-hatch supply of these fatty acids. These results may have implications in diet of pregnant, or lactating women and the newborn infant. The current intake of omega-3 fats does not meet the recommended intake in this population. Long chain PUFA (especially DHA) is needed for neural growth and development especially during the last trimester of pregnancy and in the first two yr of post-natal life in humans when brain growth and maturation is at its peak [22, 23]. Intense accretion of long chain *n-3* PUFA such as DHA has been reported in human brain during the last trimester of pregnancy [23]. A similar pattern of high long chain PUFA accretion during the third trimester of incubation has been reported in studies using avian models [2, 3]. However, differences in nutrient requirements, metabolism, and tissue growth velocity should also be considered before extrapolating results in animal model to humans.

### *In ovo* exposure to ***n-3*** and ***n-6*** fatty acids and its impact on chick brain PUFA status

**Fig. 3:**
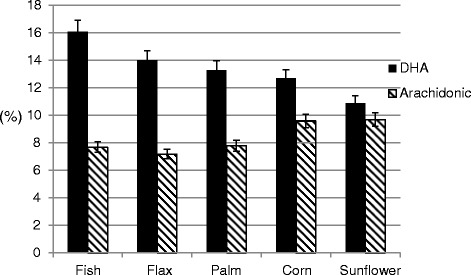
Brain docosahexaenoic (DHA) and arachidonic acid content of chicks hatched from hens fed a different lipid sources*. See Table 1 Foot note for details on diet composition

There are two sources of omega-3 fats in poultry diets. ALA which is derived from plant-based oils or oil seeds while long chain PUFA (e.g., EPA, DPA, DHA) that are derived from marine oil or algae. Long chain *n-3* and *n-6* PUFA such as DHA and arachidonic acid are abundant in the central nervous system of avians and constitute over 15 and 10 % of total lipids in newly hatched chicks [24]. It has been shown that chicken embryo preferentially accumulates DHA and arachidonic acid in the brain during the last wk of incubation [25] as observed in the human infants during the last trimester of gestation [23, 26]. Hatching egg was used as a model to test the effect of maternal diet on brain PUFA composition. To test this, different sources of oil (e.g., fish oil, flax, palm, corn or sunflower oil) were added to hen diets (3–3.5 %) and fertilized eggs were incubated. It was observed that chick brain PUFA composition reflected maternal diet. However, DHA was more sensitive to *in ovo* fatty acid manipulation than arachidonic acid (Fig. 3). Although linseed oil provided ALA (the parent *n-3* fatty acid precursor), the DHA was lower than those chicks from hens fed fish oil.

### Chick brain DHA: effect of dietary α-linolenic acid vs. docosahexaenoic acid

**Fig. 4:**
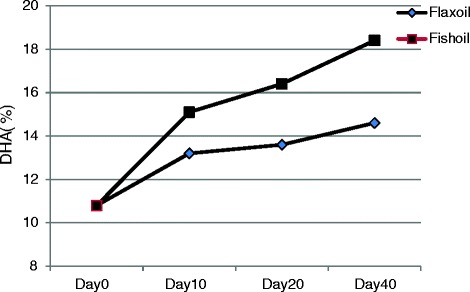
Post-hatch changes in the docosahexaenoic acid (DHA) in the brain tissue of broiler chickens. The chicks hatched from eggs depleted of DHA and were fed either flax oil or fish oil after hatch.**The breeder hen diets provided 16.0 % CP, 3.6 % calcium and 2,728 kcal metabolizable energy/kg. Sunflower oil (3.5 %) was used in breeder hen diet to produce DHA-depleted eggs and chicks. Flax oil or fish oil was included in broiler chick diet at 3.5 % and tocopherol content of the diet was 48.3 μg/g

The accretion of DHA during embryogenesis occurs from maternal sources (egg yolk) and during post hatch period through chick starter diet, similar to maternal plasma (gestation), breast milk or infant formula (postnatal) in the human infant. To test the efficacy of ALA vs. DHA in post hatch diet on maintaining brain DHA, *n-3* PUFA depleted eggs were incubated. Hatched chicks were fed either flax seed oil (ALA) or fish oil (DHA). The brain tissue DHA was assessed up to 40 days of growth. Although hens desaturated and elongated ALA, the brain DHA in flax seed oil fed chicks was lower than that of fish oil-fed chicks (Fig. 4). These results may have implication in the diets of lactating women consuming only plant-based *n-3* fats (e.g., vegetarians) or those consuming a typical high *n-6* Western diet. The diet of pregnant or nursing women in western countries is low in long chain *n-3* fatty acids with a wide ratio of *n-6*:*n-3* fatty acids, and infants are fed formulae deficient in DHA [22]. In addition, the post-natal synthesis of long chain PUFA from C18 precursors is negligible during the first four months following birth [26]. Therefore, a low supply of long chain *n-3* PUFA from maternal source (human breast milk) and infant formulae may result in low fetal and neonatal accretion of DHA with possible impairment in brain growth or development.

### *In ovo* lipid nutrition and impact on mediators of inflammation in progeny chicks

Cell membrane phospholipids are rich in long chain PUFA. Among the different PUFA, ester-linked arachidonic acid and EPA in phospholipids are potentially biologically active precursors and can be mobilized by phospholipase A2 to generate the free arachidonic and EPA which then can act as substrates for cyclooxygenase or lipoxygenase which produces eicosanoids. Eicosanoids derived from arachidonic acid such as LTB4, PG2, and TX2 are pro-inflammatory, and eicosanoids derived from EPA (e.g., LTB5, PG3,TX3) are less inflammatory [16]. To investigate whether tissue *n-3* or *n-6* PUFA status affects eicosanoid production, cells or tissues were taken at day of hatch, 7, 14 and 21 from chicks hatched from *n-3* enriched or depleted eggs. It was observed that on the day of hatch, chicks from *n-3* PUFA enriched eggs had the lowest liver and serum interleukin (IL-6), cardiac PGE_2_, TXA_2_ concentrations upon exvivo challenge [20, 10]. The effect of maternal diet persisted up to day 7 in the cardiac tissue eicosanoid concentrations (17). Similarly, LTB_4_ production by thrombocytes from *n-3* depleted chicks was greater than those chicks hatched from *n-3* enriched eggs [18]. The significant difference in LTB_4_ production in progeny birds persisted up to 21 day of bird growth. In addition, the ratio of LTB5 to LTB4 concentrations was higher in chicks hatched from *n-3* PUFA enriched eggs. The ratio of LTB5 to LTB4 was significantly correlated to the ratio of EPA to arachidonic acid in spleen and bursa in these chicks. These results indicate that an integral relationship exists between early dietary exposure to *n-3* or *n-6* PUFA through egg on tissue/cell fatty acid content, and consequently production of inflammatory mediators in progeny chickens.

### *In ovo* lipid nutrition: impact on immune responses in progeny chicks

Inflammation is part of the chick’s immediate response to challenges (e.g., infection) and is part of the normal innate immune responses. However, when inflammation occurs in an uncontrolled or inappropriate manner, it can affect production performance or disease progression. In chicks, developmental events important for immuno-competence are initiated during the embryonic period and continue in the early weeks following hatching [27]. Therefore, targeting towards a robust immune system during early post-hatch may enhance chick quality and health. Hatching through first week of life is the most vulnerable period affecting early mortality and culls. During this time, the chick faces abrupt and profound metabolic, physiological and environmental stressors. These peri- and early post-hatch stressors in chicks are contributed through: shifting from chorio-allantoic respiration to pulmonary respiration with resulting exposure to atmospheric oxygen and increase in rate of oxidative metabolism; transition from yolk lipid-based metabolism to solid carbohydrate-based metabolism through the diet; the long gap in hatching time (>24 h), delays in shipments to farms leading to early starvation. In addition, other parental factors such as breeder hen age and nutrition and environmental conditions of the farm can affect health and quality of the newly hatched chick [2, 3]. Chick quality and survivability during early post hatch period depend upon their ability to respond effectively, appropriately and timely to these different stressors.

The impact of an early supply of *n-3* and *n-6* PUFA through hatching eggs in modulating humoral and cell mediated immune responses in the hatchlings was investigated. One group of humoral mediators that accomplish the humoral immune responses is antibodies. The immunoglobulin G (IgG) is the major class of antibodies produced during humoral response and is the primary antibody circulating in chick’s blood. Hatching eggs from hens fed diets containing sunflower oil (linoleic, *n-6*) or linseed oil (ALA) at different ratios were collected and incubated. It was observed that hatchlings from hens fed the diets containing linoleic:ALA of 12.4:1 showed lower bovine serum albumin-specific IgG titer in the serum than those chicks from hens fed diet containing linoleic:ALA of 0.8:1. Newly hatched chick is heavily reliant on maternally produced antibodies (passive immunity) for its own immune defense before it becomes immunocompetent, which generally takes about 2 weeks. All maternal Ig needed to protect hatching chicks must be present in the egg and transported from the yolk across the yolk sac into the circulation of the developing chicks. In this context, these results suggest that changes in linoleic:ALA in the egg affects the passive immunity of hatching chicks [28]. Similarly, delayed hypersensitivity test (DTH) response was suppressed (~50-fold) in 14 and 28-days-old chicks hatched from eggs with high *n-3* PUFA [29]. Overall, these results provide the evidence that *in ovo* supply of *n-3* PUFA has an effect on passive immunity in progeny chicks which may extend up to over 50 % of post-hatch life. A summary of the research reported on the role of early exposure to essential *n-3* or *n-6* fatty acids and its impact on different immune responses is reported in Table 2. Understanding the biological mechanisms underlying in ovo exposure to lipids through hatching eggs offers an exciting opportunity to apply this knowledge in developing feeding strategies to improve post-hatch chick immune health and productivity.

**Table 2:** Summary of studies investigating the effect of early exposure of lipids and its impact on progeny chickens immune or inflammatory responses

References	Lipids in maternal diet	Reported findings in the progeny during growth
Bullock et al., 2014 [20]	Fish oil vs. sunflower	Chicks from fish oil-fed hens had the lower liver and serum IL-6 concentrations than those from sunflower-oil fed hens.
Gonzalez et al., 2011 [36]	Fish oil vs. sunflower	Early access to *n-3* PUFA increased the expression of COX-2: actin ratio in lipopolysaccharide injected birds.
Bautista-Ortega et al. 2009 [17, 35]	Sunflower vs. Fish oil	Prostaglandin E_2_ concentration in cardiac tissue was higher in one day-old chicks from hens fed sunflower oil than those from fish oil.
Cherian et al. 2009 [35]	Sunflower vs. Fish oil	Prostaglandin E_3_ and thromboxane A_2_ production by peripheral blood mononuclear cells was reduced in 7-day old chicks from hens fed low *n-3* (0 % DHA) vs. high *n-3* (4.2 % DHA).
Hall et al., 2007 [18]	Sunflower vs. Fish oil	Ratio of LTB_5_ to LTB_4_ remained higher up to day 21 in chicks hatched from high omega-3 (4.1 %) vs. low omega-3 (0.9 %) eggs.
Wang et al. 2002, 2004 [28]	Sunflower and Linseed oil	High linoleic:ALA (12.4:1 vs. 0.8:1) led to lower BSA-specific IgG titer in the serum in the hatchlings Feeding breeder hens 5 % fish oil diet decreased BSA-induced wing web swellings at 4 week of age in chicks.
		Chicks from the hens fed linseed and fish oil diet had lower splenocyte and thymus lymphocyte proliferative response than those from sunflower-oil fed chicks.
Liu and Denbow 2001 [21]	Soybean oil, Chicken fat, Menhaden oil	Maternal dietary *n-3* fatty acids lowered the *ex vivo* prostaglandin E2 production of tibiae in newly hatched quail compared to those from hens fed soybean oil or poultry fat.

### *In ovo* lipid nutrition: impact on hatchling antioxidant status

Modern strains of birds selected for fast growth have high metabolic rates and increased oxidative stress. Antioxidant capability at hatching time is considered to be an important determinant of chick viability. Antioxidants in the animal body work together as the so called “antioxidant system” to prevent damaging effects of free radicals and toxic products of their metabolism. The chick’s antioxidant system includes enzymes (e.g., superoxide dismutase, glutathione peroxidase, glutathione reductase, and catalase) and molecules (e.g., glutathione, vitamin A and E, and carotenoids) [30, 31]. Antioxidants are needed to protect chicks from oxidative damage. Chick nutritional encephalomalacia is a classical vitamin E-deficiency syndrome characterized by a severe hemorrhagic lesion of the cerebellum resulting in ataxia and death [32]. Experimental induction of nutritional encephalomalacia in chicks fed high PUFA diet attests to the unique need of antioxidants such as vitamin E in providing protection against oxidative damage [33]. Hatching time is considered to be a period of high oxidative stress due to long-chain PUFA accretion in tissues, exposure to atmospheric oxygen, onset of pulmonary respiration, and sudden increase in rate of oxidative metabolism [3] and the hatchlings are expected to react with a compensatory induction of endogenous antioxidants. The impact of hatching egg *n-3* PUFA content on antioxidant status was assessed. It was observed that chicks hatched from hens fed fish oil had the lowest level of liver vitamin E content compared to flax or sunflower oil [33]. Liver tissue super oxide dismutase and glutathione peroxidase activity were highest in chicks hatched from hens fed fish oil [34, 35]. These results suggest that regulation of antioxidant activity in newly hatched chicks are dependent on parent hen diets and egg PUFA composition.

## Summary

The decreased age to market of modern-day commercial broiler chicken has increased the importance of nutrition during the early pre and post-hatch period. Currently, little consideration is given to the composition of the breeder hen dietary fat composition and what effect it may have on reproduction or the immune or inflammatory response in progeny birds. The chick embryo relies on nutrients deposited by the hen in the egg for sustaining over one-third its life. Early exposure to lipids and essential *n-3* or *n-6* fatty acid through hatching egg can influence cell membrane fatty acids, the production of inflammatory mediators and antioxidant status. Lipids act both directly (e.g., by replacing arachidonic acid vs. EPA as an eicosanoid substrate) and indirectly (e.g.*,* by altering eicosanoid generation, expression of inflammatory proteins/genes) and are summarized in Table 3 [36]. It is becoming increasingly clear that early exposure to lipids and essential fatty acids has metabolic effects due to provision of energy during embryonic growth. The influence of *in ovo* fatty acid exposure may extend through the entire production phase for broilers. Therefore, feeding the embryo or “early diet manipulation” offers a powerful and holistic tool to promote the health of hatchlings in a natural way. The information derived through *in ovo* feeding will expand our knowledge of early nutrition, and can lead to dietary strategies that will ameliorate hatchability loss, culls and early chick mortality. Furthermore, considering the uniqueness of hen and the fertile egg where embryo develops outside the host, properly designed experiments in hens fed well-controlled diets can facilitate new and innovative comparative nutrition research, expanding our knowledge of maternal diet and early nutrition in other non avian systems.

**Table 3:** Overall effects of early exposure of omega-3 lipids through hatching egg in progeny chicks

• Increase in tissue cell membrane *n-3* fatty acids
• Increase in production of less pro-inflammatory eicosanoids
• Decrease in long chain *n-6* PUFA in cell membrane phospholipids
• Decrease in production of pro-inflammatory eicosanoids
• Decrease in production of interleukin-6
• Decrease in cell mediated immune response measured through DTH response
• Reduced splenocyte and thymus lymphocyte proliferative response
• Reduction in hatched chick body weight
• Reduction in hepatic tocopherol content
• Increase in liver tissue SOD and GSH-PX activity
• Alteration in the expression of inflammatory COX-2 proteins
• Alteration in the expression of genes related to lipid metabolism

## Abbreviations

ALA: α-Linolenic acid
PUFA: Polyunsaturated fatty acid
EPA: Eicosapentaenoic acid
DPA: Docosapentaentoic acid
DHA: Docosahexaenoic acid
YSM: Yolk sac membrane
VLDL: Very low density lipoprotein
DTH: Delayed type hypersensitivity
TX: Thromboxane
PG: Prostaglandin
LT: Leukotriene
PBMN: Peripheral Blood Mononuclear
